# The Influence of Shift Work on the Quality of Sleep and Executive Functions

**DOI:** 10.5334/jcr.194

**Published:** 2020-06-24

**Authors:** Mojtaba Elhami Athar, Mohammad-Kazem Atef-Vahid, Ahmad Ashouri

**Affiliations:** 1 School of Behavioral Sciences and Mental Health (Tehran Institute of Psychiatry), Iran University of Medical Sciences, Tehran, IR

**Keywords:** executive functions, quality of sleep, shift work, day work

## Abstract

Shift work is an inconsistent and atypical work schedule. This study aimed to investigate the influence of shift work on the Quality of Sleep (QOS) and Executive Functions (EF). Thirty shift workers and thirty day workers first completed a demographic questionnaire. They then were tested using the Pittsburgh Sleep Quality Index (PSQI) and EF tests, including the Corsi Block-Tapping Task (CBTT), Berg’s Card Sorting Task (BCST), and the Continuous Performance Task (CPT). Results were subjected to non-parametric Chi-Square Tests, the Mann-Whitney U Test, and Independent T-Tests. Shift workers had significantly poorer sleep quality than day workers, which was shown in PSQI global scale (*p* = 0.001), sleep duration (*p* = 0.042), habitual sleep efficiency (*p* = 0.021), and sleep disturbance (*p* = 0.021). Concerning EF tests, shift workers performed significantly poorer on CBTT (*p* = 0.019) and BCST (*p* = 0.015, 0.047) compared with day workers. Significant differences were also observed between shift workers and day workers in terms of variables of omission errors (*p* = 0.037) and commission errors (*p* = 0.041) on CPT, but no significant difference was found between shift workers and day workers in reaction time (*p* = 0.561). Shift work impaired EF. These findings are related to shift workers’ poorer sleep and its detrimental effects on areas of the brain, which are critical for EF, such as the prefrontal area. Our results suggest the evaluation and implication of practices and policies to assuage the consequences of working in shifts.

## Introduction

Shift work is an inconsistent and atypical work schedule, compared to the typical daytime work schedule [1]. It refers to work schedules outside of usual daytime hours and includes evening, night, morning, rotating, and irregular shift schedules [2,3]. Several studies have demonstrated various adverse effects of unusual working hours [4,5,6]. Regarding the deleterious effects of shift work on the essential brain functions, most of the studies have investigated its consequences on cognitive functions [7,8,9]. However, only a few research studies have investigated the effect of shift work on executive functions (EFs). Executive functions are a set of general-purpose controlling mechanisms associated with the prefrontal lobe, which control the dynamics of human actions and cognition [10]. Core components of EF are cognitive flexibility/shifting, response inhibition, and working memory [11].

Numerous studies have shown the deleterious effects of poor sleep on those areas of the brain that have a critical role in EF, such as the prefrontal cortex (PFC), the thalamus, and the connectivity between the frontal network and the limbic system [12,13,14,15,16]. Impairments in EF disturb everyday life performance by decreasing the ability to adjust to environmental demands [17]. It is also associated with long-term vocational and psychosocial disturbances and a decreased quality of life [18,19,20]. Additionally, since EF and cognitive functions – processes involved with input, storage, processing, and output of information- work concomitantly, an impairment in EF can disturb cognition [21]. Since shift work is associated with poor sleep, shift workers may experience issues mentioned above, thereby decreasing the safety at works [14,22]. All these taken into account, it can be concluded that intact EFs are crucial for the normal performance of workers.

Machi et al. [23] reported the impairment of cognition due to shift work in Career Emergency Physicians and concluded that short-term memory was the cognitive function most affected by both day and night shifts. Marquire et al. [8] indicated the association of shift work with a chronic impairment of cognition, which was stronger for exposures lasting more than ten years. In another study by Shah et al. [24] on medicine rotating residents, the authors reported a trend towards poorer scores on nights versus days in processing time and response time, but no differences were found for attention and memory capabilities. Other similar studies have found that shift workers had poorer performance on threat detection [25], a sustained attention task (PVT) [26], and sensory memory [27].

In addition to EF, sleep quality is a variable susceptible to shift work because working in shifts results in interrupted sleep, problems in going to sleep, and early wakening the combination of which decreases shift workers’ quality of sleep [28]. Also, poor sleep quality decreases cognition, along with some aspects of health and well-being [29].

The authors’ literature review revealed no study that specifically investigated the influence of shift work on EFs. In the present study, the authors aimed to fill this gap in the literature by investigating the effect of shift work on the quality of sleep and EFs.

## Participants and Methods

### Participants and Procedure

The present study was conducted with shift workers (N = 30) and day workers (N = 30) of Tabriz University security staff. Both groups had a similar working environment, organizational structure, and same job duties. Because of the low number of female workers in both groups, only male workers were included in the study. Therefore, this study focuses on male shift workers and day workers. The day workers’ work schedule included five days of consecutive work starting at 07:00 h and ending at 15:00 h, followed by two rest days. In contrast, the shift workers’ work schedule was 24 hours work and 48 hours off. Also, the shift work schedule was a rotating shift, which included consecutive work for 4 hours followed by two rest hours repeated for 24 hours during which they were allowed to take naps. The exclusion criteria for participating in this study included brain injury, alcohol and/or substance abuse, use of hypnotic drugs, and history of psychiatric illness. The present study was evaluated and confirmed by the ethical committee of the Iran University of Medical Sciences (IUMS) (approval number: IR.IUMS.FMD.REC 1396.9411556002). The participants were first informed of the aims and process of the study. They were then informed about the confidentiality of the collected data and that the data would solely be used for the present study. After the participants had given their informed consent to participate in the study, the research began. The participants first completed a demographic questionnaire and PSQI. Then they were administered the EF tests (through freely accessible software package- The Psychology Experiment Building Language) [30], which included The Corsi Block-Tapping Task (CBTT), Berg’s Card Sorting Task (BCST), and Continuous Performance Task (CPT), respectively, which were administered in a quiet room under the supervision of a research assistant. Data were collected from all of the participants during the mornings of the days that they started to work, so day differences for the participants were controlled. Moreover, it took about 35 minutes for each participant to complete all the tasks.

#### Instruments

##### The Pittsburgh Sleep Quality Index (PSQI)

The PSQI was developed by Boysse et al. [31]. It measures sleep quality and disturbances based on seven component scores, including subjective sleep quality, sleep latency, sleep duration, habitual sleep efficiency, sleep disturbances, use of sleeping medication, and daytime dysfunction. These components are calculated on a scale of 0 to 3 and then are summed to yield a global PSQI score ranging from 0 to 21, with higher scores indicating poorer sleep quality [31]. In the present study, the PSQI was mainly used to compare two groups, regarding the quality of sleep and the sleep deprivation, which measured by the PSQI’s “sleep duration” component.

##### Corsi Block-Tapping Task (CBTT)

The Corsi Block-Tapping Task is one of the most practical tools for the assessment of visuospatial short-term memory; it is a span task and a visuospatial analogue to the digit span as an index of verbal short-term memory [21]. In this task, a staggering array of nine colored squares is displayed. A sequences of squares lights up, and participants click on the squares to repeat the sequence. Participants complete two practice trials followed by twelve test trials: two trials per sequence length starting with three squares and continuing to eight squares [30].

##### Berg’s Card Sorting Task (BCST)

Berg’s Card Sorting Task (BCST) is a computerized version of the WCST (Wisconsin Card Sorting Test) provided by the Psychology Experiment Building Language (PEBL) version 0.13 [30]. The BCST makes use of a card deck containing 128 cards, each card containing a different combination of one of four shapes, colors, and quantities. Four key cards are displayed at the top of the screen as a guide to help determine to which of the four stacks the deck’s up-card is to be sorted. The deck is revealed one card at a time, and the visible card is matched to key cards depending on the particular rule (unknown to the participant) for a given set. The set is completed after ten cards have been successfully matched (i.e., the participant has acquired the rule for the first attentional set). The sorting rule is then changed (again unknown to the participant). The new rule must be discovered using trial and error and through feedback received after each card is sorted. After a card is sorted, the participant is provided with feedback regarding whether it was sorted correctly (i.e., according to the current rule). This process continues until the participant either sorts all 128 cards or until the participant successfully completes 9 sets/categories. The BCST takes approximately 10 min to complete [32]. The present study included: 1-the primary measure of the BCST, the number of” perseverative errors “, which occur when the individual continues to sort cards according to a previous, but no longer relevant or correct sorting rule, and 2- the number of completed categories.

##### Continuous Performance Task (CPT)

The Continuous Performance Task (CPT) of the Psychology Experiment Building Language (PEBL) system [30] was used. The English alphabet letters of A, B, C, D, E, F, G, H, I, J, L, M, O, P, Q, R, S, U, and X were randomly presented, one at a time, in the center of the computer screen, with all letters except ‘X’ being the target for the response. In the task, participants respond by pressing the spacebar of a computer when any target letters appeared. Letters appeared at intervals of 1,000, 2,000, and 4,000 ms. Twenty stimuli were created by dividing 360 stimuli into 18 blocks. Each stimulus was randomly presented 3 times with intervals between each presentation. Each letter appeared for 200ms. The task takes about 14 minutes [33]. The analyses involved three variables: omission errors, commission errors, and response time.

### Statistical analyses

In the present study, for data entry and statistical analyses, SPSS 20 software was used. Normal distribution suitability tests confirmed that the data were appropriate for the use of parametric and non-parametric tests. Chi-square test was used to compare descriptive variables of the groups, including age, marital status, and level of education. The Mann-Whitney U Test and Independent T-Test were used to compare EFs of the two groups. It was decided beforehand that a P level of less than 0.05 would be accepted as indicating statistically significant results.

## Results

The mean age of the shift worker group was 34.93 (SD = 5.26 years), ranging from 27 to 46 years. Similarly, the mean age of the dayworker group was 36.37 (SD = 7.66 years), ranging from 25 to 53 years. The majority of participants had an undergraduate degree (46.7% of shift workers, 40% of day workers), and the majority of both groups were married (96.7% of shift workers, 86.7% of day workers). The differences between the groups in term of age (X^2^ = 1.673, P = 0.643), marital status (X^2^ = 1.964, P = 0.161), and education (X^2^ = 1.404, P = 0.705), were not statistically significant. Thus, the groups were matched with regard to these variables. For more details see Table 1.

**Table 1:** Comparison of Day and Shift workers on demographic variables.

Variables	Type of work		Total (n = 60)	X^2^	P

	Shift workers (n = 30)	Day workers n = 30)			

Age (%)					
20–30 years	8 (26.7)	8 (26.7)	16 (26.7)	1.673	0.643
31–40 years	18 (60)	15 (50)	33 (55)		
41–50 years	4 (13.3)	6 (20)	10 (16.7)		
50<	0 (0)	1 (3.3)	1 (1.7)		
Marital status (%)					
Married	29 (96.7)	26 (86.7)	55 (91.7)	1.964	0.161
Unmarried	1 (3.3)	4 (13.3)	5 (8.3)		
Education (%)					
Diploma	9 (30)	7 (23.3)	16 (26.7)	1.404	0.705
Associated Diploma	5 (16.7)	7 (23.3)	12 (20)		
Undergraduate	14 (46.7)	12 (40)	26 (43.3)		
Graduate	2 (6.7)	4 (13.3)	6 (10)		

*Note*: * p < 0.05

### Quality of Sleep

The quality of sleep and its components (measured by PSQI) were compared between the groups by using the Mann-Whitney U test. Results showed that there were significant differences between the groups in PSQI global scale (p = 0.001), sleep duration (p = 0.042), habitual sleep efficiency (p = 0.021), and sleep disturbance (p = 0.021). (Table 2).

**Table 2:** Comparison of shift and day workers on PSQI.

Variables	Shift workers (n = 30)		Day workers (n = 30)		Z-Score	P	

	Mean/S.D.	Median	Mean/S.D.	Median			

PSQI global score	6.60/3.223	6.50	4.43/2.473	4	–3.223	0.001*
Subjective sleep quality	1.13/0.860	1	0.83/0.531	1	–1.285	0.199
Sleep latency	1.23/1.135	1	0.83/0.913	1	–1.332	0.183
Sleep duration	1.47/0.937	1.50	0.97/0.809	1	–2.029	0.042*
Habitual sleep efficiency	0.57/0.728	0	0.23/0.568	0	–2.302	0.021*
Sleep disturbance	1.23/0.679	1	0.83/0.592	1	–2.299	0.021*
Use of sleep medication	0.0	0	0.0	0	0.00	1
Daytime dysfunction	1/1.050	1	0.73/0.785	1	–0.809	0.419

*Note*: * p < 0.05

### Executive functions test results

There were significant differences between the two groups on various measures of EF. Regarding the Visuospatial Working Memory task, assessed by the CBTT, the result of a Mann-Whitney U test (p = 0.019) showed a significant difference between the two groups with shift workers performing poorer than the day workers (Table 3).

**Table 3:** Comparison of shift and day workers on Corsi Block-Tapping Task (CBTT).

Groups	Sample	Mean	SD	Median	Z-score	P

Shift workers	30	4.93	0.70	5	–2.354	0.019*
Day workers	30	5.45	0.66	5		

*Note*: * p < 0.05

The performance on BCST was significantly better for day workers compared to shift workers. Day workers made significantly fewer perseverative errors than shift workers (t = 2.49, p = 0.015). Also, the number of categories completed by day workers was significantly more than shift workers (t = –2.03, p = 0.047).

On CPT, day workers performed better than shift workers; day workers performed significantly better than shift workers on omission errors (t = 2.15, p = 0.037) and commission errors (t = 2.09, p = 0.041). Regarding reaction time, the difference was not statistically significant (t = –0.58, p = 0.561). Detailed results are presented in Table 4.

**Table 4:** Comparison of the groups on Berg’s Card Sorting Task (BCST) and Continuous Performance Task (CPT).

Variables	Shift workers (n = 30)		Day workers (n = 30)		T-Value	P	

	Mean	S.D.	Mean	S.D.			

BCST						
Perseverative error	29.70	11.23	23.40	8.03	2.49	0.015*
Completed categories	4.40	2.343	5.57	2.09	–2.03	0.047*
CPT						
Omission error	9.20	9.15	5.20	4.36	2.15	0.037*
Commission error	18.60	7.25	14.83	6.67	2.09	0.041*
Reaction time	397.55	50.05	404.68	44.33	–.0.58	0.561

*Note*: * p < 0.05

## Discussion

The present study focused on the influence of shift work on the quality of sleep and executive functions (EFs). The results indicated that shift workers had poorer sleep quality and performed significantly poorer on EF tasks, as measured by The Corsi Block-Tapping Task (CBTT), Berg’s Card Sorting Task (BCST), and Continuous Performance Task (CPT). These results are discussed below.

Previous studies have suggested that the impairment of shift workers’ cognition could result from circadian misalignment, sleep deprivation, and fatigue [34]. The limitation of the current study was that the authors did not measure the participants’ circadian misalignment and fatigue. Future studies are needed to better operationalize the measure of these factors in order to investigate their influence on shift workers’ EF. But results of PSQI and its sleep duration component (which measures sleep quantity) showed that shift workers had significantly lower sleep hours than day workers, so they were sleep-deprived. This result is in line with related studies. In a study by Escriba, Perez-Hoyos, and Bolumar [28], the authors found empirically that shift work reduced shift workers’ length of sleep (by 2 hours for permanent shift workers and by 30 minutes for workers on rotational working systems). It also led to poor sleep quality. Shift workers suffer from insufficient sleep, and since they try to sleep during the day, they cannot have the quality of sleep enjoyed by night sleepers; therefore, shift workers have overall poorer sleep quality [35]. Additionally, shift work is associated with difficulty in sleeping, intermittent sleep, and early wakening, all of which decrease shift workers’ quality of sleep [28].

Several studies have indicated the negative effects of sleep deprivation and poor sleep on certain brain areas. Neuroimaging studies using PET and FMRI techniques have revealed the detrimental effects of sleep deprivation on PFC and thalamus areas, which have a crucial role in executive functions [13].

The findings of the current study regarding the poor performance of shift workers in visuospatial working memory compared to day workers are in line with previous studies regarding the influence of sleep deprivation on working memory [36,37]. Sleep deprivation might affect spatial working memory by reducing hippocampal AMPA receptors [36]. Sleep loss alters the relative expression levels of AMPA and NMDA receptors; thus, affecting the synaptic strength and capacity for brain plasticity as well as partially contributing to spatial working memory impairment [37].

Results on cognitive flexibility, which we measured by BCST, revealed that shift workers had significantly poorer performance than day workers. The ability to categorize based on different criteria and conceptual reasoning were lower in shift workers. Neuroimaging studies have confirmed that performance on BCST is associated with PFC [38]. Complex cognitive functions such as cognitive flexibility mainly rely on PFC, which is sensitive to sleep deprivation [14]. Glucose metabolism of PFC decreases after sleep deprivation and does not entirely reverse even following one night of recovery sleep [15,16]. This decrease in the metabolism of PFC is likely to underlie shift workers’ poor performance in BCST. Furthermore, the poor performance of shift workers on BCST is probably due, in part, to rigid thinking, which results from sleep deprivation [39].

Regarding CPT, shift workers had significantly more omission and commission errors compared to day workers. The analyses compared the sustained attention of the groups according to the number of omission errors, defined as lapsing or failing to respond to a presented stimulus. Other studies support the results of the present study concerning sustained attention. In a study by Valdez, Ramírez, García, Talamantes, and Cortez [40], participants adhered to a 28-hour constant routine protocol during which feeding, room temperature, motor activity, and room illumination were controlled to be continuously stable. After 28 hours of constant sleep deprivation, sleepiness and fatigue increased while indices of sustained attention (measured by the CPT), continuously declined. They hypothesized that the results seemed to be due to the accumulation of sleep pressure with time awake. Sleep deprivation also decreases neural activity in PFC (a region critical for alertness and sustained attention), thereby disturbing sustained attention [16,41]. Finally, Lim and Dinges recognized vigilant attention as a cognitive process that is consistently and strongly vulnerable to sleep deprivation [42].

Concerning response inhibition, the authors compared the number of each group’s commission errors defined as the ability to inhibit inappropriate responses [43]. In CPT, response inhibition is measured by the number of responses given to non-targets (i.e., Xs). The poorer performance of shift workers during response inhibition is in line with evidence from the literature [12,43,44,45]. The poor performance of shift workers during response inhibition could be attributed to disturbed functioning of the prefrontal region of the brain as a result of sleep deprivation, which shift workers experience. Also, the deleterious effects of chronic stress on neuropsychological functions such as mental speed, learning, and memory are significant [45]. Sleep deprivation lowers inhibition and enhances impulsivity to negative stimuli. Response inhibition depends on the integration of diverse frontal loci, which is vulnerable to sleep loss [12]. Further, the connectivity between frontal networks and the limbic system is diminished as a result of sleep deprivation [12].

Concerning the reaction time, the results showed no statistically significant difference between the groups. A review of the literature revealed contradictory results in this regard. The results of two studies demonstrated the negative influence of shift work on the reaction time of medical residents [24,46]. The medical residents in shift work schedules had impaired reaction time compared with the control group [24,46]. The result of the present study regarding reaction time is supported by studies of Kazemi et al. [47] and Namita, Ranjan, and Shenvi [48]. A possible explanation for the result of the present study regarding reaction time is that perhaps shift workers adapted to reduced sleep after working continuously in shifts [48]. The possibility of “resetting” circadian rhythms has been discussed among researchers.

The importance of sleep for EF is well documented in several studies [49,50]. Shift work impairs the normal sleep of workers. In such a situation, they suffer from a poor sleep quality, difficulty in falling asleep, daytime sleepiness, sleep deprivation, and poor physical and mental performance. Thus, individuals in rotational shift work have to constantly battle the detrimental effects of shift work [51]. Shift work is associated with a wide range of consequences for workers and jobs. Sleep deprivation reduces concentration, learning ability, motor skills, and slows reaction time [52]. It also decreases communication skills and the ability to cope with workplaces’ emotional demands. Sleep deprivation impairs decision-making and increases risk-taking behaviors [53]. A number of environmental disasters in the past few decades, such as Three Mile Island nuclear plant incident, the Chernobyl explosion, and the space shuttle challenger explosion, have been partly attributed to human errors due to sleepiness and fatigue [34]. Similarly, more than half of the nurses who worked in shifts reported being sleep deprived and made more patient care errors [54].

## Conclusion

The results of this study showed the presence of a significant decrease in sleep quality and impairments in the EF of the shift workers, including visuospatial working memory, cognitive flexibility, sustained attention, and response inhibition. These findings are related to shift workers’ poorer sleep and its detrimental effects on areas of the brain that are critical for EF, such as the prefrontal area. Disturbances in EF can increase risks of accidents at work and the number of human errors [22,34]. Considering our findings and possible consequences of impairments in EF we suggest preventive measures. Previous studies have shown that exposure to bright light can increase shift workers’ performance on working memory and concentration [55]. Additionally, while the benefits of a short night nap have a limited impact, longer naps improve cognitive performance for a longer time, and thereby it can act as a beneficial ergonomic strategy [56].

## Future Research

The present study solely investigated the EFs of male shift workers. A study that investigated predominately female shift workers, for example, nurses, would be informative in terms of understanding any gender differences in this area because gender may affect EF performance. The present study focused on rotating shift work, thereby future research may be informative regarding the impact on EF of the different types of shift work. Finally, while the association between intelligence (defined as IQ) and EF has been demonstrated in previous studies [57], and the education levels of participants in the present study suggest that they were of at least normal intelligence, it would be informative to study intelligence’s influence on the relationship between shift work and executive functions.

## Note

The information contained in this article was extracted from MEA’s master’s thesis in the School of Behavioral Sciences and Mental Health (Tehran Institute of Psychiatry), Iran University of Medical Sciences, Tehran, Iran.
